# Editorial: Assessing and evaluating the psychosocial impact of the Covid-19 pandemic on anxiety and stress: perspectives from the Indian sub-continent

**DOI:** 10.3389/fpsyt.2024.1389515

**Published:** 2024-04-25

**Authors:** S. M. Yasir Arafat, Sujita Kumar Kar, Rakesh Singh, Russell Kabir

**Affiliations:** ^1^ Senior Research Fellow, Biomedical Research Foundation, Dhaka, Bangladesh; ^2^ Department of Psychiatry, King George’s Medical University, Lucknow, UP, India; ^3^ Department of Research, Transcultural Psychosocial Organization Nepal, Kathmandu, Nepal; ^4^ School of Allied Health, Faculty of Health, Medicine, and Social Care, Anglia Ruskin University, Chelmsford, United Kingdom

**Keywords:** Covid-19, pandemic, Indian subcontinent, anxiety, stress

The world has started healing from the impact of the Covid-19 pandemic. It has affected the mental well-being of people all over the world and stressed the mental healthcare delivery system significantly in a variety of ways. The psychological issues associated with Covid-19 were significant and there was high perceived need for mental healthcare by general public during the initial phase of pandemic, when the information and understanding about Covid-19 was poor (1). Across the pandemic there is exponential growth in research evidences regarding the association of psychological issues with Covid-19 pandemic. One recent umbrella review identified that the prevalence of anxiety symptoms varied from 24.4% in general populations to 41.1% for vulnerable groups, depression varied from 22.9% in general populations to 32.5% for vulnerable groups, and stress-related symptoms was 39.1% (2). High-income countries with a resilient health system felt threatened to cope with the burden. On the other hand, countries with low- and middle-income category (LMIC) like countries in Indian Subcontinent (Bangladesh, Bhutan, India, Maldives, Nepal, Pakistan, and Sri Lanka) struggled a much while facing the pandemic due to death, physical morbidity, services burden during the pandemic. One recent scoping review identified that the prevalence of anxiety, depression, and stress widely varies based on the study methods, study settings, phase of the pandemic and instrument used to assess the measures (3). It found the prevalence of anxiety varied from 2.5% in North India to 53% in Bangladesh, depression varied from 3.5% in North Indian slum to 29.8% in Pakistan, and stress related symptoms varied from 18.3% in Pakistan to 59.7% in Bangladesh (3).

The goal of this Research Topic was to explore the impact of the Covid-19 pandemic on anxiety and stress on the population of the Indian Sub-continent. We aimed to focus on a unique opportunity to explore possible cross-cultural differences in the expression of anxiety and stress in these times, and to study anxiety and stress in relation to the Covid-19 pandemic.

The Research Topic included four studies in both qualitative and quantitative study designs. In one study Hitch and Zaman described the experiences of Covid-19 pandemic among students both Black, Asian and Minority Ethnic (BAME) and White groups in the UK through in-depth interviews. The authors found that mental health problems were more common among female students, specially among the BAME female students. There was an increase in social media engagement, lack of personal space was noted among BAME females students and reduction in personal hygiene among the White female students.

In another qualitative study, Parvaresh-Masoud et al. assessed the coping strategies of the first responders i.e. emergency medical technicians (EMTs) of Covid-19 pandemic to promote mental health and well-being during the pandemic. The study found four primary coping methods mentioned as seeking social support, ensuring self-care, using the coping mechanisms, and finding aims in the work in response to the stress while dealing Covid-19 infected patients.

A study by Kalrao et al. assessed the parenting stress and related factors among healthcare professionals during the second wave of Covid-19 pandemic in India. The study revealed that 23% of healthcare professionals had severe parenting stress measured by the parenting stress scale. The study also found that female gender, providing care for more than two weeks, working spouse in Covid-19 pandemic care, having a child with behavioural problems, and living in joint family were associated with high parenting stress. Lastly, the other study was conducted in Dhaka, Bangladesh by Kibria et al. assessed the rate and associated factors of depression and anxiety among Covid-19 survivors. The study revealed that the prevalence of depressive symptoms was 26% and anxiety symptoms was 23.2% in the Covid-29 survivors. The study also found that old age (≥60 years), and comorbidities were associated with both depression and anxiety while hospitalization for Covid-19 infection was associated with anxiety.

To conclude the articles collected in this Research Topic provide valuable insights about the psychosocial impact of Covid-19 pandemic on anxiety and stress in a densely populated region with LMIC background. It contributes to revealing the regional as well as global impact of Covid-19 pandemic measured as stress and anxiety.

## Author contributions

SA: Writing – review & editing, Writing – original draft. SK: Writing – review & editing. RK: Writing – review & editing. RS: Writing – review & editing.
